# A Case of Tubulointerstitial Nephritis With Uveitis Diagnosed From Isolated Glucosuria Detected During School Urinary Screening

**DOI:** 10.7759/cureus.77447

**Published:** 2025-01-14

**Authors:** Naonori Kumagai, Mami Akamatsu, Yoshiki Kawamura, Haruo Mizuno, Yohei Ikezumi

**Affiliations:** 1 Department of Pediatrics, Fujita Health University School of Medicine, Toyoake, JPN; 2 Department of Pediatrics, Fujita Health University Okazaki Medical Center, Okazaki, JPN

**Keywords:** dipstick, glucosuria, low-molecular-weight protein, school urinary screening, tubulointerstitial nephritis with uveitis, β2-microglobulin

## Abstract

We report a case of tubulointerstitial nephritis with uveitis (TINU) diagnosed from isolated glucosuria detected during school urinary screening.

The patient was a 12-year-old girl in whom glucosuria was detected during school urinary screening using a dipstick; however, urinary protein and occult blood were negative. There were no preceding symptoms of infection or medication. The patient visited the Fujita Health University Okazaki Medical Center two weeks after the school urinary screening for further examination. No edema or skin rash was observed. A urine test showed urinary glucose was positive and urinary β2-microglobulin was high; other values were almost normal. Mild renal dysfunction was observed. There was no hyperglycemia or high HbA1c level; therefore, diabetes mellitus was ruled out. Various autoantibody tests were negative, and the angiotensinogen-converting enzyme level was within the normal range. The patient was clinically diagnosed with idiopathic tubulointerstitial nephritis without a renal biopsy. Renal dysfunction tended to improve gradually after the first visit. Three months after the first visit, conjunctival congestion appeared in the right eye, and the patient was diagnosed with uveitis and eventually with TINU.

When performing detailed examinations for urinary glucose, it is necessary to differentiate kidney disease as well as diabetes mellitus. Moreover, it is necessary to recognize that even if the urine dipstick test is negative for protein, it may be positive for low-molecular-weight protein.

## Introduction

In Japan, school screening for urinary protein and occult blood using a dipstick was started in 1974 for the early detection of kidney disease [1], and urinary glucose was added in 1994 for the early detection of diabetes mellitus [2]. If the patient's urine tests positive for protein or occult blood, a detailed examination for kidney disease is performed, and if the urine tests positive for glucose, a detailed examination for diabetes mellitus is performed.

Tubulointerstitial nephritis with uveitis (TINU) is a syndrome consisting of idiopathic tubulointerstitial nephritis and uveitis. Usually, these two conditions do not occur simultaneously [3]. It may be asymptomatic or present with nonspecific symptoms such as fever, weight loss, rash, joint pain, and abdominal pain [3,4]. Renal dysfunction and tubular protein are observed; however, a significant albuminuria is not usually observed [3,4]. Moreover, proximal tubular dysfunction, such as glycosuria, aminoaciduria, and metabolic acidosis, is often observed [3].

Herein, we report a case of TINU diagnosed from isolated glucosuria detected during school urinary screening.

## Case presentation

Glucosuria was identified in a 12-year-old girl during school urinary screening using a dipstick; however, urinary protein and occult blood tests were negative. There were no preceding symptoms of infections or medications. She visited the Fujita Health University Okazaki Medical Center two weeks after the school urinary screening for further examination. She was 151.9 cm tall, weighed 47.1 kg, and had no edema or skin rash. Hematological test results upon the first visit (Table 1) were not significant. 

**Table 1:** Hematological test results upon the first visit

Peripheral blood		
Red blood cells	4100000	/μL
Hemoglobin	10.8	g/dL
Hematocrit	34.7	%
Platelets	311000	/μL
White blood cells	6100	/μL

Blood chemistry test results upon the first visit (Table 2) showed no hyperglycemia or elevated HbA1c levels; therefore, she was not diagnosed with diabetes mellitus. A mild decrease in the creatinine-estimated glomerular filtration rate (Cr-eGFR) at 83.06 mL/min/1.73 m^2^ was observed. The angiotensinogen-converting enzyme (ACE) level was within the normal range (reference range 8.3-21.4 U/L). 

**Table 2:** Blood chemistry test results upon the first visit

Blood chemistry		
Total bilirubin	0.3	mg/dL
Glutamic-oxaloacetic transaminase	26	U/L
Glutamic-pyruvic transaminase	23	U/L
Lactate dehydrogenase	154	U/L
Alkaline phosphatase	163	U/L
Creatine kinase	106	U/L
Blood urea nitrogen	15.9	mg/dL
Creatinine	71	mg/dL
Uric acid	3	mg/dL
Total protein	7.8	g/dL
Albumin	3.7	g/dL
Sodium	140	mEq/L
Potassium	4	mEq/L
Chloride	107	mEq/L
Calcium	9.4	mg/dL
Inorganic phosphorus	3.7	mg/dL
C-reactive protein	0.16	mg/dL
Angiotensinogen-converting enzyme	10.5	U/L
Glucose	91	mg/dL
HbA1c	6	%
pH	7.317	
pCO_2_	51.4	mmHg
HCO_3_^-^	26.3	mmol/L
Base excess	-0.4	mmol/L
Anion gap	8.5	mmol/L
Lactic acid	7	mg/dL

Urinary test results (Table 3) revealed the following: urinary glucose was positive, urinary protein was trace, urinary β2-microglobulin (β2-MG) was high, and red blood cells were absent. Urinary amino acids were not measured.

**Table 3:** Urinary test results upon the first visit Urinary protein +- refers to protein levels at 15-30 mg/dL. Occult blood - refers to hemoglobin levels at 0.06-0.15 mg/dL. Urinary glucose 1+ refers to glucose levels at 100-250 mg/dL. Urinary ketone body - refers to ketone levels at 0-10 mg/dL. HPF: high-power field Cr: creatinine

Urinalysis		
pH	7	
Gravity	1.023	
Protein	+-	
Occult blood	-	
Glucose	1+	
Ketone body	-	
Red blood cell	1>	/HPF
Protein/creatinine	0.191	g/gCr
N-acetyl-beta-glucosaminidase	15.2	U/L
Beta-2 microglobulin	11630	μg/L

Immunological test results (Table 4) were unremarkable; various autoantibody tests were negative, and anti-streptolysin O antibody was not measured. 

**Table 4:** Immunological test results upon the first visit ANA: antinuclear antibody; anti-dsDNA antibody: anti-double-stranded deoxyribonucleic acid; anti-SS-A antibody: anti-Sjogren's antibody A; anti-SS-B/LA antibody: anti-Sjogren's antibody B/LA; PR3-ANCA: proteinase3 antineutrophil cytoplasmic antibody; MPO-ANCA: myeloperoxidase antineutrophil cytoplasmic antibody; anti-GBM antibody: anti-glomerular basement membrane antibody; IgG: immunoglobulin G; IgA: immunoglobulin A; IgM: immunoglobulin M;

			Reference range
ANA	40>		40>
Anti-dsDNA antibody	2.4	IU/mL	10>
Anti-SS-A antibody	1>	U/mL	10>
Anti-SS-B/LA antibody	1>	U/mL	10>
PR3-ANCA	1>	U/mL	3.5>
MPO-ANCA	1>	U/mL	3.5>
Ant-GBM antibody	2>	U/mL	3.0>
CH50	54.7	CH50/mL	25.0 - 48.0
C3c	111	mg/dL	73 - 138
C4	25	mg/dL	11 - 31
IgG	2144	mg/dL	
IgA	166	mg/dL	
IgM	121	mg/dL	

**Figure 1:**
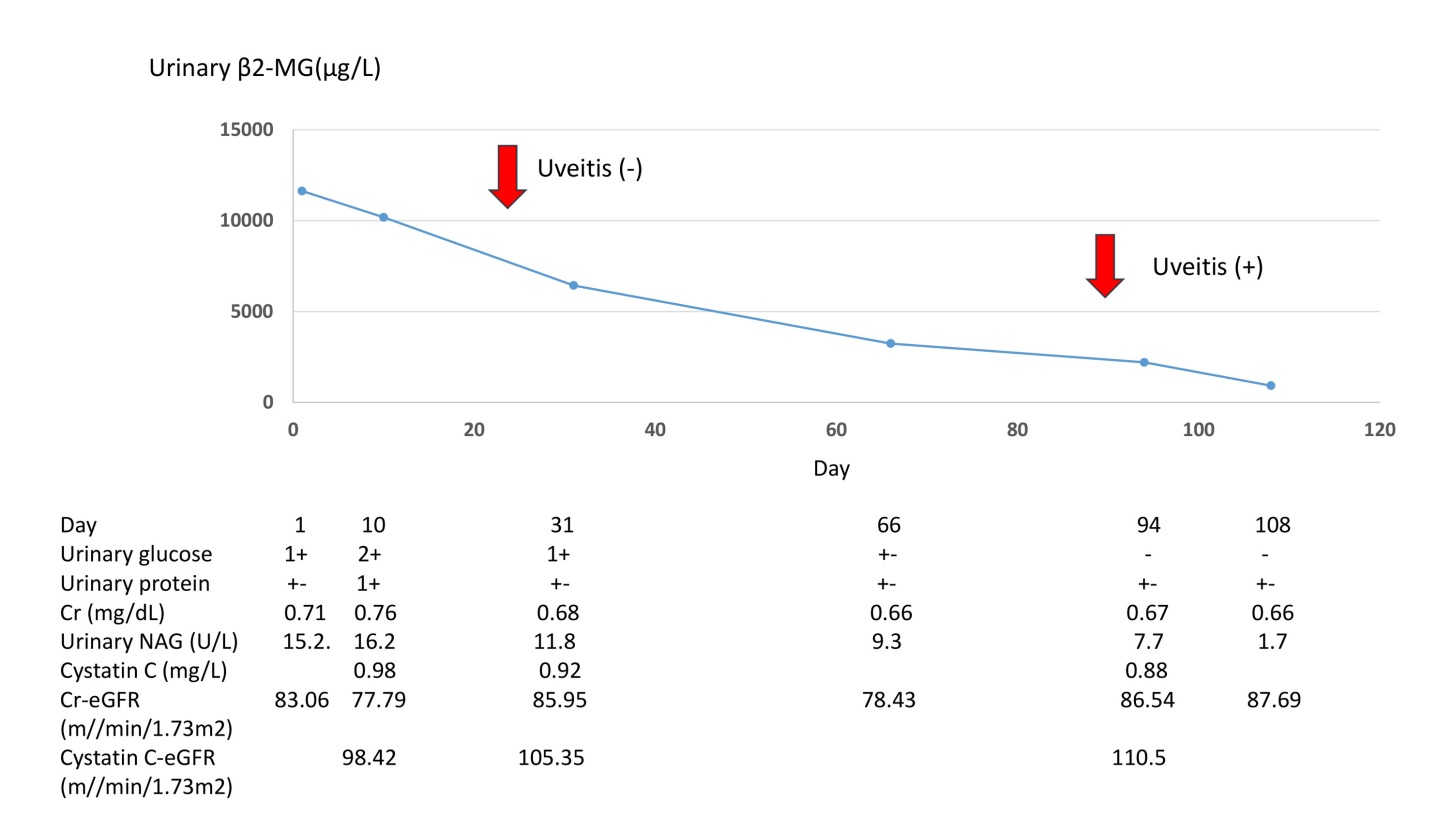
Clinical course of the patient Renal dysfunction tended to recover gradually after the first visit. Three months after the first visit, conjunctival congestion appeared in the right eye. Urinary glucose -, +-, 1+, and 2+ refer to glucose levels at 0-50, 50-100, 100-250, and 250-500 mg/dL, respectively. Urinary protein +- and 1+ refer to protein levels at 15-30 and 30-100 mg/dL, respectively. β2-MG: beta-2 microglobulin; Cr: creatinine; NAG: N-acetyl-beta-glucosaminidase; eGFR: estimated glomerular filtration rate;

Renal ultrasound did not reveal any renal enlargement; however, it showed a slight increase in the brightness of the renal parenchyma. A renal biopsy was not performed since renal dysfunction tended to recover gradually after the first visit (Figure 1) and the patient was clinically diagnosed with idiopathic tubulointerstitial nephritis. Glucosuria was determined to be caused by proximal tubular damage resulting from tubulointerstitial nephritis. An ophthalmologic examination performed three weeks later did not reveal uveitis. Three months after the first visit, conjunctival congestion appeared in the right eye, and the patient was diagnosed with uveitis. Since uveitis coexisted with tubulointerstitial nephritis in the same patient, the patient was eventually diagnosed with TINU.

## Discussion

In the present patient, isolated glucosuria was detected during school urinary screening using a dipstick, and the patient's urine tested negative for protein and occult blood. The patient was not diagnosed with diabetes mellitus despite thorough investigations. She had renal function impairment, mild urinary protein, and a significantly elevated urinary β2-MG level, leading to the diagnosis of tubulointerstitial nephritis. There were no preceding symptoms of infection or drug use, various autoantibody tests were negative, and the ACE level was within the normal range, suggesting that the patient's tubulointerstitial nephritis was idiopathic. Therefore, a renal biopsy was not performed, and the patient's diagnosis of idiopathic tubulointerstitial nephritis was based solely on clinical findings. Three months after the first visit, the patient was diagnosed with uveitis due to congestion in the right eye, and TINU was eventually diagnosed. When glucosuria is detected during school urinary screening, diabetes mellitus and renal glucosuria are examined in detail; however, kidney disease may not be adequately ruled out [5]. To the best of our knowledge, this is the first case of a patient diagnosed with TINU based on isolated glucosuria identified during school urinary screening as a renal condition. Moreover, as a kidney disease diagnosed from the isolated glucosuria identified during school urinary screening, a case of juvenile nephronophthisis has previously been reported [6]. In this previous case, urinary screening performed using a dipstick revealed isolated urinary glucose positivity as urinary protein was negative; however, the urinary β2-MG level was high, just like in the present case. When the urinary glucose is positive and the urinary β2-MG level is high simultaneously, proximal renal tubular dysfunction is suspected, making it easier to diagnose kidney disease. In Japan, school urinary screening is performed using a dipstick, making it difficult to detect low-molecular-weight proteins such as β2-MG, since a dipstick can hardly detect low-molecular-weight proteins [7]. Even if only urinary glucose is positive, it is important to differentiate kidney disease carefully by measuring the urinary β2-MG level, since it may be caused by kidney disease that requires treatment and careful follow-up as in the present case.

In this patient, renal dysfunction and uveitis were not observed simultaneously as the former preceded the latter. The patient was initially diagnosed with idiopathic tubulointerstitial nephritis; however, three months after the first visit, congestion appeared in the right eye, and uveitis was diagnosed, leading to the diagnosis of TINU. In 65% of cases of TINU cases, renal dysfunction precedes uveitis, and eye symptoms appear an average of 3-14 months after renal dysfunction. In 15% of the cases, renal dysfunction and uveitis occur simultaneously, and in 20% of the cases, uveitis precedes renal dysfunction [3]. Uveitis is often initially unilateral [3]. In the present patient, renal impairment was indicated as the initial manifestation based on the abnormal urinalysis findings, and unilateral uveitis developed three months after the onset of renal dysfunction, indicating the typical clinical course. In TINU, renal dysfunction and ocular manifestations are rarely observed simultaneously at the first visit; therefore, when either uveitis or renal dysfunction is observed, careful follow-up is necessary to check for the appearance of renal dysfunction or eye symptoms up to 12 months to differentiate TINU. In the present patient, tubulointerstitial nephritis was clinically diagnosed without a renal biopsy, which is usually required to make a definitive diagnosis of tubulointerstitial nephritis [3,8]. However, it is also considered that a renal biopsy should be performed in cases of severe or persistent renal dysfunction or atypical progression; otherwise, a clinical diagnosis is preferable [8]. In the present patient, tubulointerstitial nephritis was suspected based on mild renal dysfunction, mild urinary protein, an elevated urinary β2-MG level, and urinary glucose positivity, and the renal impairment and tubular dysfunction tended to improve gradually; therefore, the patient was clinically diagnosed with tubulointerstitial nephritis without a renal biopsy. When tubulointerstitial nephritis is suspected, it is important to decide whether to perform a renal biopsy for diagnosis based on the patient's clinical status.

## Conclusions

We report a case of TINU diagnosed based on isolated glucosuria detected during school urinary screening. When performing detailed examinations for urinary glucose, it is necessary to differentiate kidney disease from diabetes mellitus and to recognize that even if the urinary dipstick test is negative for protein, low-molecular-weight protein may still be present in the urine. When tubulointerstitial nephritis is diagnosed, it is important to consider TINU as a differential diagnosis and undergo repeated ophthalmological examinations even if there are no eye symptoms.
